# Initial Tendon Retraction is Associated with Muscle Degeneration After Nonoperatively Treated Proximal Hamstring Avulsions

**DOI:** 10.1186/s40798-026-01024-x

**Published:** 2026-05-06

**Authors:** Sofia Laszlo, Anne-Mari Rosenlund, Elsa Pihl, Målfrid Holen Kristoffersen, Olof Sköldenberg, Jörg Schilcher, Martin Eklund, Frede Frihagen, Mikael Skorpil, Kenneth B. Jonsson

**Affiliations:** 1 Department of Surgical Sciences, Unit of Orthopedic Surgery, Uppsala University, Uppsala University Hospital, 75185 Uppsala, Sweden; 2 Division of Orthopaedic Surgery, Oslo University Hospital, Oslo, Norway; 3 Institute of Clinical Medicine, Faculty of Medicine, University of Oslo, Oslo, Norway; 4 Department of Clinical Sciences at Danderyds Hospital, Karolinska Institutet, Stockholm, Sweden; 5 Department of Orthopedic Surgery, Haukeland University Hospital, Bergen, Norway; 6 Department of Orthopedic Surgery, Faculty of Health Sciences, Linköping University, Linköping, Sweden; 7 Department of Biomedical and Clinical Sciences, Faculty of Health Sciences, Linköping University, Linköping, Sweden; 8 Department of Medical Epidemiology and Biostatistics, Karolinska Institutet, Stockholm, Sweden; 9 Department of Orthopaedic Surgery, Østfold Hospital Trust, Grålum, Norway; 10 Department of Molecular Medicine and Surgery, Karolinska Institutet, Stockholm, Sweden

## Abstract

**Background and Purpose:**

Magnetic resonance imaging (MRI) is essential in assessing proximal hamstring avulsions (PHA), where tendon retraction is a key pathological consequence. Beyond its diagnostic role, MRI may also provide prognostic information on subsequent muscle degeneration. This study aimed to determine whether pre-treatment MRI findings, particularly tendon retraction, predict later muscle degeneration and clinical outcomes in nonoperatively treated PHA patients.

**Methods:**

This study is a post hoc analysis of nonoperatively treated patients (*n* = 95) with complete proximal hamstring avulsions from the Proximal Hamstring Avulsion Clinical Trial (PHACT). Diagnostic MRIs were reassessed for tendon retraction, Wood classification, number of tendons avulsed, and hematoma size. The primary outcome was muscle degeneration, defined by the loss of lean muscle volume (LMV) and an increase in muscle fat fraction (MFF) quantified by DIXON MRI at 24 months. The secondary outcome was maximum hamstring muscle isometric force at 24 months. Outcome data was expressed as the limb symmetry index (LSI), which was the measurement of the injured hamstring expressed as a percentage of measurement of the uninjured hamstring. Linear regression was used to analyze the association between diagnostic MRI measurements, patient factors, and LSIs for LMV, MFF, and maximum isometric force.

**Results:**

The median (IQR) LSIs of the LMV, MFF and maximum strength were 78% (67.2, 87.1), 139% (124.5, 166.8), and 84% (75.4, 94.0), respectively, at 24-month follow-up. A multivariate linear regression model including tendon retraction, age, sex, hematoma size and whether the dominant limb was injured explained 48%, 48% and 23% of the variance in the LSIs of LMV, MFF and maximum force, respectively. Tendon retraction was the strongest explanatory factor for the variance of muscle degeneration observed in patients with nonoperatively treated PHA.

**Interpretation:**

Greater initial tendon retraction is associated with increased muscle atrophy and fat infiltration in the hamstring muscles in patients with nonoperatively treated PHA.

*Trial registration*: NCT03311997.

**Supplementary Information:**

The online version contains supplementary material available at 10.1186/s40798-026-01024-x.

## Key Points

- MRI-based measures of tendon retraction and hematoma size were independently associated with the extent of muscle degeneration after proximal hamstring avulsion.
- Muscle degeneration occurred even with small retractions, indicating a retraction-dependent relationship with structural change.
- Associations between muscle morphology and strength were moderate, suggesting that neuromuscular and functional factors also influence recovery.

**Supplementary Information:**

The online version contains supplementary material available at 10.1186/s40798-026-01024-x.

## Introduction

Proximal hamstring avulsion (PHA) is a severe injury commonly associated with slip and fall incidents in daily activities and sports [1–3]. Magnetic Resonance Imaging (MRI) is the gold standard for the diagnosis of PHA [3–5] and for determining the number of avulsed tendons and degree of tendon retraction. In addition, edema, displacement of the sciatic nerve, and size of the hematoma can be visualized. MRI findings, along with a clinical examination and patient characteristics, guide treatment decisions among clinicians [6, 7]. Although the clinical significance and prognostic value of tendon retraction have not been thoroughly studied, retraction above 2 cm is typically considered to support surgical intervention [1, 3, 7, 8]. Only one previous publication has explored the association of pre-treatment MRI findings with clinical outcomes after nonoperative treatment of PHA, finding a moderate association between initial tendon retraction and patient outcomes [9].

Results from the only randomized controlled trial comparing operative and nonoperative treatment (the Proximal Hamstring Avulsion Clinical Trial, PHACT) [10]demonstrated that nonoperative treatment is noninferior to operative treatment, as measured by patient-reported outcomes at 24 months. These results challenge the traditional preferences for operative treatment and are supported by recent cohort studies [11–13] that fail to demonstrate a benefit of surgery. Data from PHACT indicate that some degree of hamstring muscle degeneration is inevitable. However, identifying patients at higher risk of excessive degeneration could help guide more individualized treatment decisions.

The aim of this study was to assess whether diagnostic MRI findings, such as tendon retraction and hematoma size, and patient factors at the time of injury are associated with muscle degeneration after 24 months in patients with acute PHA managed nonoperatively. We hypothesized that these factors would be associated with the extent of muscle degeneration, defined by loss of lean muscle volume and increased fat fraction, and with isometric strength at follow-up.

## Materials and Methods

### Trial Design

This is a post hoc analysis of nonoperatively treated individuals in PHACT [10]. PHACT was a multicenter, preference-tolerant, randomized controlled noninferiority trial carried out across ten hospitals in Sweden and Norway [10, 14]. Ethical approval was obtained from the Uppsala Regional Ethical Committee and the Regional Committee of Medical and Health Research Ethics in Norway.

### Participants

**Fig. 1:**
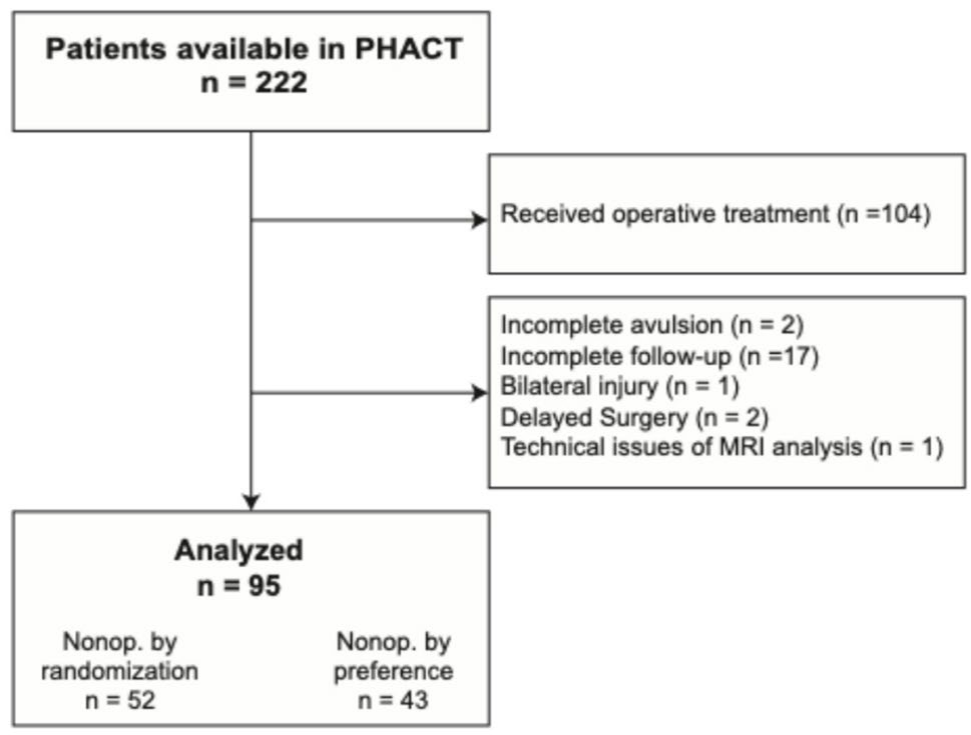
Study flowchart. PHACT is the proximal hamstring avulsion clinical trial. Incomplete follow-ups were missing MRIs at 2 years. Delayed surgery were patients initially treated nonoperatively but operated on before the follow-up MRI. Bilateral injury refers to one patient with a prior contralateral hamstring avulsion, excluded because side-to-side comparison was not possible

Patients, 30–70 years of age with a suspected PHA were screened for inclusion in PHACT [10]. Eligibility required an active lifestyle and MRI confirmation of an acute (within 4 weeks) proximal avulsion of at least two hamstring tendons. Exclusions encompassed patients with multiple injuries and an unacceptable surgical risk. Participants were randomly assigned to operative reattachment of the tendons or nonoperative treatment. In cases where either the patient or the surgeon exhibited a clear preference for a particular treatment, inclusion in an observational cohort was offered. Recruitment started October 2017 and concluded in July 2020. The current study focused on the nonoperatively treated patients within both the randomized trial and the observational cohort of PHACT, with a retrievable diagnostic MRI and 24-month follow-up. Only patients with complete avulsions involving all proximal hamstring tendons were included, while patients with incomplete injuries and patients operated on prior to the follow-up MRI were excluded (Fig. 1). Baseline data on age, sex, body mass index (BMI) and footedness were collected. The side of injury and footedness were combined into a single binary variable indicating if the dominant limb was injured.

### Diagnostic MRI

Patients underwent MRI scans following local clinical protocols at participating hospitals for diagnosing suspected hamstring avulsion injuries, in this study referred to as diagnostic MRIs. Imaging protocols varied, encompassing sequences such as proton-density (PD), PD fatsat, PD SPAIR (spectral attenuated inversion recovery), PD Dixon, T2 fatsat, T2 Dixon, STIR (short tau inversion recovery), TIRM (turbo inversion recovery magnitude), T2-weighted, and T1-weighted. Despite varying sequences and scan parameters, all clinical protocols were designed to detect hamstring avulsion injuries, and since the proximal hamstring tendon is hypointense on all sequences, retraction measurements could be performed consistently. The scans encompassed the origin of the hamstring muscles at the pelvis and extended through one or both thighs. The initial interpretation of the MRI images was conducted in accordance with local protocols.

Diagnostic MRIs were independently re-evaluated by a musculoskeletal radiologist (Skorpil) and an orthopedic resident (Laszlo). Injuries were classified according to the Wood classification [1], and the number and identity of avulsed tendons were determined. Tendon retraction was measured using the method described by van der Made [15]. This involved identifying the center point of the proximal hamstring origin on the upper region of the ischial tuberosity on coronal images and measuring the shortest distance from this point to the most proximal part of the hypointense tendon stump (Fig. S1). Hematoma size was quantified by examining all axial images to find the axial plane having the largest hematoma and then measuring the largest diameter (Fig. S1). In cases of differing assessments between the two evaluators, the case was discussed until consensus was reached.

### Outcome Variables

#### MRI at 24-Month Follow Up

The primary outcome was muscle degeneration defined by the loss of lean muscle volume (LMV) and increase in muscle fat fraction (MFF) of the injured hamstrings in relation to the uninjured hamstrings, as measured on DIXON MRI at the 24-month follow-up. A detailed description of the MRI scan protocol and the subsequent analysis chain at AMRA (AMRA Medical, Linköping, Sweden), image processing, segmentation-methods, and quality control measures of the service have been previously published [10, 16–19]. In summary the hamstring muscles, including the semimembranosus, semitendinosus, and both the short and long heads of the biceps femoris, were fully segmented bilaterally. The total muscle volume (TMV), LMV and MFF of each individual muscle were calculated. The TMV was defined as the sum of all voxels included in a muscle segment, and MFF as the total volume of fat within the muscle segment divided by the total muscle volume. LMV was defined as TMV * (1-MFF).

#### Strength Measurements

Maximum isometric force (Newton, N) was measured in both the injured and uninjured leg at 24 months. Patients were positioned supine with the hip stabilized against the examination table. A handheld isometric dynamometer (microFET 2, Hoggan health industries) was placed on a 15 cm block on the examination table. The heel of the tested leg was then placed on the dynamometer and the knee angle adjusted with a goniometer to approximately 15°. Each patient performed a 5-second maximal contraction by pressing the heel downward against the dynamometer, engaging both knee-flexor and hip-extensor components of the hamstrings. The highest value from three predefined trials was recorded. The procedure has been described in detail in the supplementary appendix of the main PHACT publication [10].

### Data Analysis

Data were analyzed using RStudio version 2023.12.0 + 369. Independent variables included patient characteristics (age, sex, BMI, dominant limb injured) and MRI findings at the time of injury (tendon retraction, Wood classification and diameter of the hematoma). Dependent variables were limb symmetry indices (LSI) of LMV, MFF, and maximum force in the injured hamstring at 24 months. Values for the injured leg were divided by those of the uninjured hamstring and multiplied by 100 to calculate LSI. The LSI thus summarized the within-subject contrast between limbs, as a representation for the change in muscle quality and function induced by the injury and served as the dependent variable in subsequent association models.

Bivariate correlations were calculated using Pearson’s correlation coefficient to assess relationships between various continuous variables. Correlation strength was categorized as follows: <0.1 (negligible), ≥ 0.1 to < 0.4 (weak), ≥ 0.4 to < 0.7(moderate), and ≥ 0.7 (strong) [20].

The association between baseline MRI findings and patient characteristics with outcomes of muscle degeneration and function was assessed using both univariate and multivariate linear regression analyses. Univariate linear regression analyses were first performed to assess the association between baseline MRI characteristics and patient factors with each outcome variable. For multivariate analysis, candidate models were generated using the Leaps R package, which systematically evaluated all possible subsets of predictor variables to identify models that optimized adjusted R². Based on this selection process, a unified linear regression model was constructed—including age, sex, BMI, injured dominant side, tendon retraction, and hematoma size—to examine their collective association with LSIs for LMV, MFF, and maximum muscle force.

Model performance was quantified using the adjusted R², Akaike Information Criterion (AIC), and Root Mean Square Error (RMSE). Predictor variables with p-values < 0.05 were considered statistically significant. In addition, the relative importance of each predictor was assessed using the varImp function from the caret R package, which yielded results identical to ranking predictors by the absolute value of their t-statistics.

Prior to modeling, missing data for the independent variables were imputed as follows: three missing BMI values were replaced with the cohort median BMI, one missing footedness value (used to determine the injured dominant side) was imputed as “injury to the dominant side”, and one missing muscle force measurement was replaced with the cohort’s median value.

## Results

### Patients

In PHACT, 118 out of 222 available patients received nonoperative treatment. Following exclusions due to incomplete follow-ups and other reasons, 95 patients remained for analysis (Fig. 1; Table 1). The median age was 53.9 years (range: 30 to 70, interquartile range [IQR]: 49 to 58) and 67% were female. Most cases were classified as Wood type 5 (complete injury with tendon retraction) (*n* = 87), with 8 cases classified as Wood type 4 (complete injury without retraction). The median tendon retraction was 4 cm, with an IQR of 2.5 to 6 cm. The median diameter of the hematoma was 4 cm (IQR: 2.5 to 6, [Table 1]).

### MRI Outcomes and Maximum Muscle Force at 24 Months

Hamstring muscle degeneration and loss of strength were evident in the injured compared to the uninjured hamstrings at 24-month follow-up. The median LSI of the LMV was 78% (IQR: 67 to 87%). The median LSI of the MFF was 139% (IQR: 125% to 167%) and the median LSI of maximum muscle force was 84% (IQR: 75 to 94%, [Table 2]).

### Bivariate Correlations

There were strong correlations between the absolute lean muscle volume and maximum muscle force at the 24-months follow-up (Table 3). This correlation was slightly stronger in the uninjured leg compared to the injured leg (Pearson correlation: *r* = 0.78 vs. 0.67). The loss of LMV, as measured by the limb symmetry index, showed a strong inverse correlation with an increase in muscle fat fraction (*r* = -0.86). The loss of LMV and increase in MFF were moderately correlated with the loss of strength (*r* = 0.54 and − 0.47). Weaker but statistically significant correlations were seen between tendon retraction, patient age, BMI, and muscle degeneration. The correlations were even weaker when tendon retraction, BMI, and age were correlated to loss of maximum muscle force at 24 months (Table 3). Among the pretreatment factors, tendon retraction showed the strongest correlation to muscle degeneration and muscle strength.

### Linear Regression Modelling

Univariate linear regression using patient and pretreatment MRI data as independent variables and the loss of lean muscle volume as the dependent variable revealed statistically significant associations for several variables (Table S1). Similar results were observed when the dependent variables were increase in muscle fat fraction and loss of maximum muscle force. Among these, tendon retraction had the strongest explanatory value for both the loss of lean muscle volume and the increase in muscle fat fraction. However, for muscle force, BMI, hematoma size, and tendon retraction showed similar explanatory strength in their individual associations (Table S1).

**Fig. 2:**
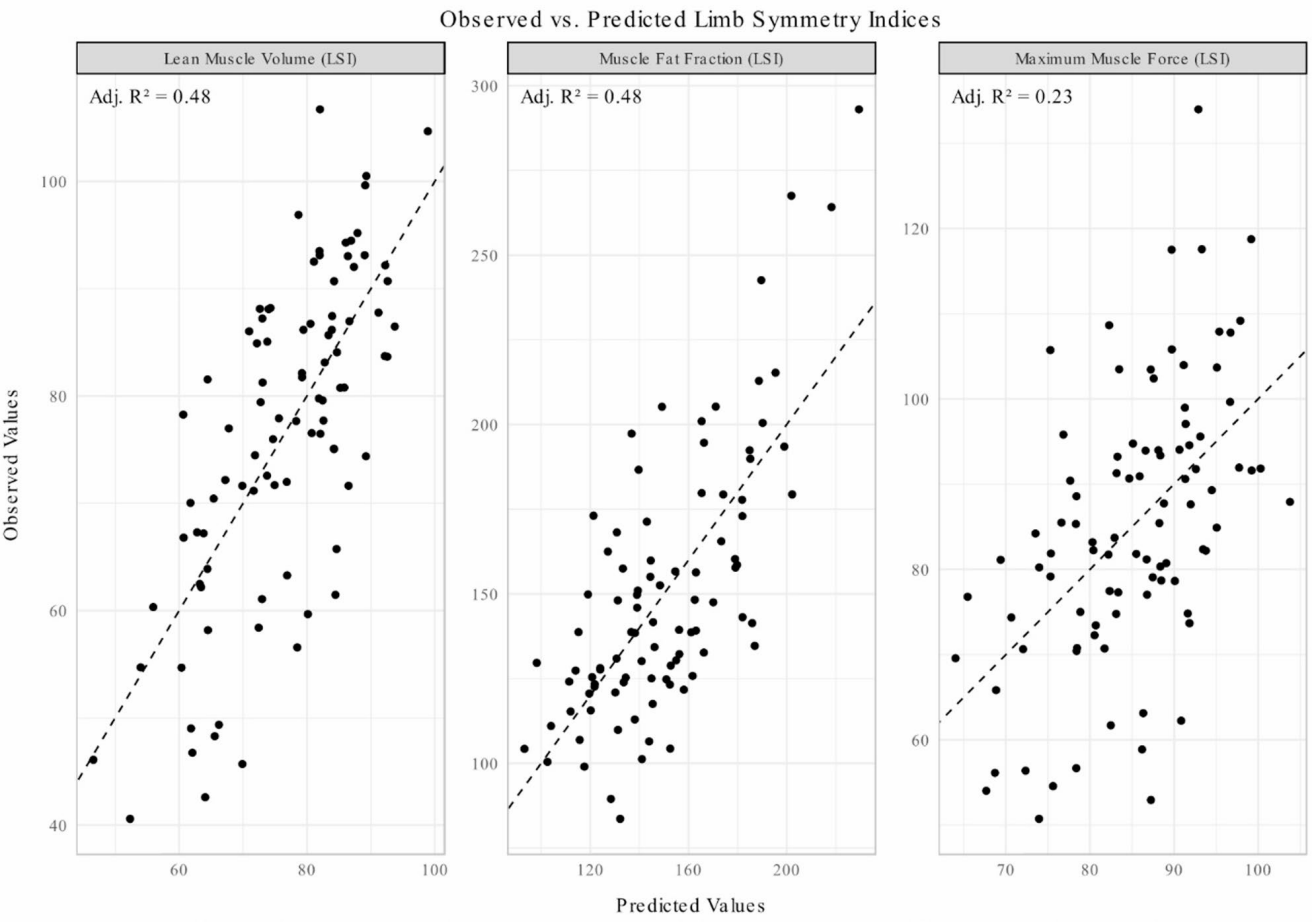
Performance of the multiple regression model across three outcomes. The scatter plots depict the observed values against the predicted values for the three dependent variables: loss of lean muscle volume, increase in muscle fat fraction, and loss of maximum muscle force. The independent variables in the model were age, sex, tendon retraction, Body Mass Index (BMI), whether the dominant limb was injured, and hematoma size. The adjusted R² values (0.48 for lean muscle volume and muscle fat fraction, and 0.23 for muscle force) indicate the proportion of variance explained by the model for each outcome

Multiple linear regression evaluated the association of pretreatment MRI data and patient factors with the outcomes; muscle degeneration and muscle force. A single, unified model—including age, BMI, sex, tendon retraction, hematoma size, and whether the dominant leg was injured—was applied to all outcomes (Table S2). This model explained 48% of the variance in the loss of LMV, 48% of the variance of the increase in muscle fat fraction, and 23% of the variance in the loss of muscle force (adjusted R-squared values: 0.48, 0.48, and 0.23, respectively; Fig. 2; Table 4). Performance metrics (Table S3) indicate that the unified model performed best for lean muscle volume, as evidenced by the lowest Akaike Information Criterion (AIC) value. Although the model achieved a similar adjusted R² for muscle fat fraction, the substantially higher Root Mean Square Error (RMSE) for that outcome suggests larger absolute prediction errors. In contrast, for maximum muscle force, the model explained less variance but yielded a relatively low RMSE, indicating more precise predictions in absolute terms.

Diagnostic tests confirmed that the model met the assumptions of linear regression. The Durbin-Watson test showed no significant autocorrelation, the Breusch-Pagan test supported homoscedasticity, and the Shapiro-Wilk test indicated that the residuals were normally distributed. Furthermore, multicollinearity was minimal (Variance Inflation Factor: 1.01–1.37; Table S4).

Tendon retraction emerged as the strongest predictor of muscle degeneration, particularly for lean muscle volume and muscle fat fraction. In contrast, for the loss of maximum muscle force, BMI, hematoma size, and whether the dominant leg was injured contributed to a similar extent as tendon retraction.

**Fig. 3:**
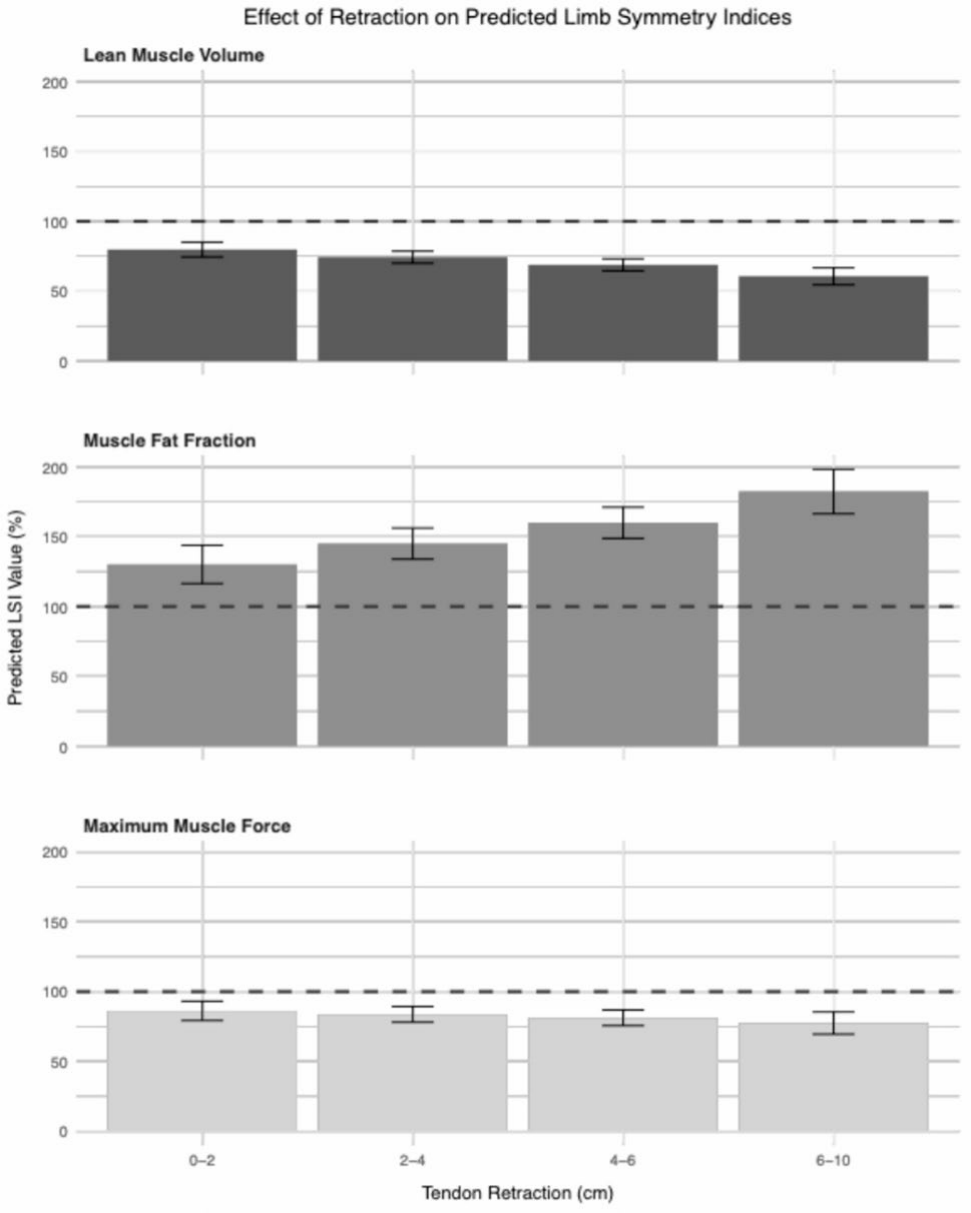
The mean marginal effect of tendon retraction in increments of 2 cm on predicted outcomes: lean muscle volume (black bars), muscle fat fraction (dark grey bars), and maximum muscle force (light grey bars). Error bars represent the 95% confidence intervals. Data are adjusted for age, gender, BMI, dominant side, and hematoma size. An LSI value of 100% indicates no difference between the injured and uninjured hamstring

To illustrate these findings, Fig. 3 shows the marginal effect of initial tendon retraction (grouped into intervals commonly used in clinical practice) on muscle degeneration and maximum muscle force.

## Discussion

Our findings demonstrate that initial tendon retraction is associated with the degree of subsequent muscle degeneration in patients with nonoperatively treated proximal hamstring avulsions (PHA). Greater retraction was significantly linked to reduced lean muscle volume and increased fat infiltration, indicating a dose-dependent relationship, where even minor retraction (< 2 cm) initiated measurable remodeling, while larger separations predicted proportionally more severe degeneration. These results highlight the predictive value of pretreatment MRI assessments, which surgeons already consider an important factor in treatment decisions [7, 21]. Reports from related injury models show that shortening or partial detachment of the musculotendinous unit can induce architectural change and fatty degeneration, supporting the concept that the hamstring muscle is highly sensitive to tension loss [22, 23].

As expected, we observed a strong correlation between lean muscle volume and muscle strength. However, the relationship between muscle degeneration and the loss of maximum isometric force was only moderate. This reflects the recognized dissociation between structural and functional recovery, as MRI measures describe morphological change, whereas strength depends on neuromuscular activation, coordination, and compensation by synergistic muscles. Furthermore, assessment of hamstring strength is influenced by the biarticular nature of the hamstrings and the contribution of synergistic knee-flexor and hip-extensor muscles. In a multicenter setting without access to computerized dynamometers, practical constraints further limit complete standardization. Although handheld devices are reliable and a uniform protocol was applied, the multicenter design likely introduced some between-site variability, which may have reduced the strength of observed associations.

Among the other covariates, body mass index was the strongest predictor of both muscle degeneration and loss of maximal force, which may reflect metabolic influences and a reduced capacity to rehabilitate with higher adiposity. Age showed a similar pattern, with older individuals exhibiting more pronounced degeneration and weakness. Hematoma size on the initial MRI also independently predicted degeneration and strength loss, even though it was only moderately correlated with tendon retraction (*r* = 0.48). Measured as the largest axial diameter, it provided a standardized, practical surrogate of bleeding extent across heterogeneous protocols and appeared to capture an additional facet of injury severity beyond retraction. A plausible explanation is that larger hematomas are accompanied by greater local edema and perineural irritation, transient mass effect, and intramuscular disruption, any of which could hinder reinnervation and recovery of normal activation, thereby contributing to secondary structural remodeling. This mechanistic interpretation remains tentative and will require dedicated studies to confirm.

Our previous randomized controlled trial (PHACT) [10] demonstrated a modest but significant protective effect of surgical reattachment on muscle degeneration. However, subgroup analysis did not show that surgery provided greater benefit for patients with more pronounced retraction when assessed by the PHAT score. Taken together, the current findings and PHACT data indicate that while tendon retraction is a strong predictor of muscle degeneration, its impact on functional and patient-perceived outcomes is less direct. Despite modest correlations with strength, tendon retraction remains an important indicator of structural injury severity and can inform individualized decisions regarding rehabilitation intensity or surgical evaluation. Focusing exclusively on nonoperatively treated patients allowed us to characterize the natural course of muscle remodeling without the confounding effects of surgery. However, the influence of surgical repair on degeneration at different levels of retraction warrants further investigation.

### Limitations of Study

Aside from the points mentioned above, our study has several limitations. Because diagnostic MRIs were obtained at multiple hospitals using local protocols, variability in scanner type and sequence settings may have introduced minor measurement error in estimating tendon retraction and hematoma size, although previous work indicates that retraction measurements are robust [15]. Quantitative baseline MRIs were not available, which precludes assessment of pre-existing asymmetries and limits true longitudinal comparison of muscle volume and fat fraction. The multicenter design also constrained the use of standardized isokinetic dynamometers for strength testing. Although a uniform handheld dynamometer protocol was applied bilaterally within each participant, complete isolation of the hamstrings is not possible in this test position, and minor variation in hip angle between individuals was unavoidable in a multicenter setting. Because both limbs were tested in identical positions within each participant, this was considered unlikely to bias limb symmetry indices. The limited number of participants made it impractical to split the data for model training and validation, which could limit generalizability. Additionally, the study cohort comprised both patients randomized to nonoperative treatment and a parallel group who actively chose this approach. While females were generally overrepresented in PHACT, this imbalance was even greater in the current study because a higher proportion of women opted for nonoperative treatment. Sex was therefore included in the multivariable model to account for potential gender-related differences in muscle degeneration.

### Strengths of Study

The prospective design and the relatively large cohort of nonoperatively treated patients enhance the validity of our findings. All imaging and strength assessments were performed according to predefined protocols, and the use of the Dixon MRI technique enabled precise quantification of lean muscle volume and fat infiltration. Centralized MRI analysis further ensured consistency across sites. Together, these factors provide a robust evaluation of muscle degeneration and functional outcomes after proximal hamstring avulsion, offering novel insight into the natural course of recovery following nonoperative treatment.

**Table 1:** Baseline characteristics

Characteristic	*N* = 95^1^
Age (years)	54 (49, 58)
Body Mass Index (kg/m^2)	25.9 (23.4, 28.4)
Unknown	3
Retraction (cm)	4.0 (2.5, 6.0)
Hematoma Size (cm)	4.0 (2.50, 6.0)
Sex	
Female	64 (67%)
Male	31 (33%)
Dominant leg injured	56 (60%)
Unknown	1
Wood type (1–5)	
4	8 (8.4%)
5	87 (92%)

^1^Median (IQR); n (%)

Pretreatment characteristics of the study population. Data are presented with Median ± Inter Quartile Range (IQR) for continuous data, and with count and percentages for categorical data. Wood is the classification of injury according to Wood et al. [1]

**Table 2:** Outcome measures

Muscle Quality	*N*	Uninjured Limb	Injured Limb	Limb Symmetry Index
Lean Muscle Volume (L)	95	0.56 (0.49, 0.71)	0.44 (0.35, 0.57)	78.3 (67.2, 87.1)
Muscle Fat Fraction	95	0.16 (0.13, 0.19)	0.22 (0.16, 0.29)	139.4 (124.5, 166.8)
Maximum Muscle Force (N)	94	176.1 (140.1, 222.5)	142.0 (121.3, 179.7)	84.0 (75.4, 94.0)

MRI muscle quality outcome measurements and muscle maximum force of the study population. Limb Symmetry index was calculated: value of injured limb/ value of uninjured limb *100. Data are presented with Median ± Inter Quartile Range

**Table 3:** Pairwise correlations

Variable 1	Variable 2	Correlation (95% CI)
Lean Muscle Volume, uninjured side	Maximum Muscle Force, uninjured side	**0.78 (0.69**,** 0.85)**
Lean Muscle Volume, injured side	Maximum Muscle Force, injured side	**0.67 (0.54**,** 0.77)**
Muscle Fat Fraction, uninjured side	Maximum Muscle Force, uninjured side	**− 0.36 (− 0.53**,** − 0.17)**
Muscle Fat Fraction, injured side	Maximum Muscle Force, injured side	**− 0.34 (− 0.51**,** − 0.15)**
Lean Muscle Volume (LSI)	Muscle Fat Fraction (LSI)	**− 0.86 (− 0.90**,** − 0.79)**
Lean Muscle Volume (LSI)	Maximum Muscle Force (LSI)	**0.54 (0.38**,** 0.67)**
Muscle Fat Fraction (LSI)	Maximum Muscle Force (LSI)	**− 0.47 (− 0.62**,** − 0.30)**
Age	Lean Muscle Volume (LSI)	**− 0.23 (− 0.41**,** − 0.03)**
BMI	Lean Muscle Volume (LSI)	**− 0.38 (− 0.54**,** − 0.19)**
Retraction	Lean Muscle Volume (LSI)	**− 0.56 (− 0.69**,** − 0.41)**
Age	Muscle Fat Fraction (LSI)	0.21 (0.00, 0.39)
BMI	Muscle Fat Fraction (LSI)	**0.44 (0.26**,** 0.59)**
Retraction	Muscle Fat Fraction (LSI)	**0.60 (0.45**,** 0.72)**
Age	Maximum Muscle Force (LSI)	− 0.09 (− 0.29, 0.11)
BMI	Maximum Muscle Force (LSI)	**− 0.34 (− 0.51**,** − 0.14)**
Retraction	Maximum Muscle Force (LSI)	**− 0.35 (− 0.51**,** − 0.15)**

Pairwise correlations between MRI-derived muscle quality parameters, maximum muscle force, and pretreatment characteristics. The upper section presents correlations between lean muscle volume (LMV), muscle fat fraction (MFF), and maximum muscle force for the injured and uninjured limbs separately. The middle section presents correlations between limb symmetry indices (LSI) of structural MRI parameters (LMV and MFF) and LSI of maximum muscle force, as well as the correlation between LMV (LSI) and MFF (LSI). The lower section presents correlations between pretreatment characteristics (age, BMI, and tendon retraction) and LSI-based outcome measures. LSI was calculated as injured limb value / uninjured limb value × 100. Statistically significant correlations are shown in bold

**Table 4:** Multiple linear regression with caret rankings

Term	Estimate (95% CI)	t - Statistic	Rank
*LSI- Lean Muscle Volume: Adjusted R*-*squared = 0.48*			
Tendon Retraction	− 2.73 (− 3.84, − 1.62)	− 4.89	1
Body Mass Index	− 1 (− 1.63, − 0.38)	− 3.18	2
Age	− 0.38 (− 0.66, − 0.11)	− 2.76	3
Dominant Side Injured (Yes)	5.92 (1.19, 10.65)	2.49	4
Sex (Male)	5.76 (0.82, 10.69)	2.32	5
Hematoma Size	− 1.49 (− 2.78, − 0.2)	− 2.29	6
*LSI-Muscle Fat Fraction: Adjusted R-squared = 0.48*			
Tendon Retraction	7.46 (4.57, 10.35)	5.14	1
Body Mass Index	2.9 (1.27, 4.53)	3.54	2
Age	0.86 (0.14, 1.58)	2.38	3
Dominant Side Injured (Yes)	− 7.1 (− 19.41, 5.21)	− 1.15	5
Sex (Male)	0.01 (− 12.83, 12.85)	0.00	6
Hematoma Size	3.14 (− 0.22, 6.51)	1.86	4
*LSI- Maximum Force: Adjusted R-squared = 0.23*			
Tendon Retraction	− 1.25 (− 2.7, 0.2)	− 1.72	3
Body Mass Index	− 0.98 (− 1.8, − 0.16)	− 2.38	1
Age	− 0.19 (− 0.55, 0.17)	− 1.04	5
Dominant Side Injured (Yes)	5.31 (− 0.86, 11.49)	1.71	4
Sex (Male)	− 1.42 (− 7.87, 5.02)	− 0.44	6
Hematoma Size	− 1.96 (− 3.64, − 0.27)	− 2.30	2

Multiple linear regression modelling of the effect of initial tendon retraction and covariates on the injury induced loss of lean muscle volume, muscle fat fraction and maximum muscle force. LSI is Limb Symmetry Index and was calculated: value of injured limb/ value of uninjured limb *100. Rank is the order of highest contribution of the independent variable to the overall performance of the model and was calculated using the varImp function in the R caret library

### Conclusions

Initial tendon retraction is a major determinant of muscle degeneration after nonoperatively treated proximal hamstring avulsions. Greater retraction is associated with more pronounced muscle atrophy and fat infiltration, whereas smaller retractions still result in measurable structural change. Although the impact of these morphological alterations on functional outcomes appears limited, this likely reflects the multifactorial nature of strength and patient-reported measures rather than an absence of biological relevance. These findings emphasize the value of detailed pretreatment MRI assessment for characterizing injury severity and supporting individualized management strategies in patients with proximal hamstring avulsion injuries.

## Supplementary Information

Below is the link to the electronic supplementary material.

Supplementary Material 1.

## Data Availability

The data and the analysis code that support the findings of this study are available from the corresponding author, upon reasonable request.

## Abbreviations

MRI: Magnetic resonance imaging
PHA: Proximal hamstrings avulsion
PHACT: The proximal hamstring avulsion clinical trial
LMV: Lean muscle volume
MFF: Muscle fat fraction
TMV: Total muscle volume
LSI: Limb symmetry index
PD: Proton density
SPAIR: Spectral attenuated inversion recovery
STIR: Short tau inversion recovery
TIRM: turbo inversion recovery magnitude
BMI: Body mass index
RMSE: Root Mean Square Error
AIC: Akaike Information Criterion
IQR: Inter quartile range
