# Supplementary material for: Initial Tendon Retraction is Associated with Muscle Degeneration After Nonoperatively Treated Proximal Hamstring Avulsions
Source: Sports Med Open. 2026 May 6;12:52. doi: 10.1186/s40798-026-01024-x (PMC13149700; doi:10.1186/s40798-026-01024-x)
Supplement: Supplementary file 1 — Supplementary Material 1. [file 40798_2026_1024_MOESM1_ESM.docx]

**Appendix**

| **Table S1. Univariate linear regression for Limb Symmetry Indices** | | | | | |
| --- | --- | --- | --- | --- | --- |
|  | **Dependent Variable** | **Term** | **Estimate (95% CI)** | **P-Value** | **R-Squared** |
|  | **Lean Muscle Volume (LSI)** |  |  |  |  |
|  |  | Tendon Retraction | -3.50 (-4.60, -2.50) | 0.00 | 0.32 |
|  |  | Hematoma Size | -3.10 (-4.40, -1.80) | 0.00 | 0.19 |
|  |  | Wood (5) | -8.60 (-19.00, 2.10) | 0.11 | 0.03 |
|  |  | Age | -0.41 (-0.77, -0.05) | 0.03 | 0.05 |
|  |  | BMI | -1.50 (-2.30, -0.74) | 0.00 | 0.15 |
|  |  | Sex (Male) | 0.86 (-5.60, 7.30) | 0.79 | 0.00 |
|  |  | Dominant Side (yes) | 3.80 (-2.30, 10.00) | 0.22 | 0.02 |
|  | **Muscle Fat Fraction (LSI)** |  |  |  |  |
|  |  | Tendon Retraction | 9.90 (7.20, 13.00) | 0.00 | 0.36 |
|  |  | Hematoma Size | 8.40 (5.00, 12.00) | 0.00 | 0.21 |
|  |  | Wood (5) | 24.00 (-3.90, 52.00) | 0.09 | 0.03 |
|  |  | Age | 0.96 (0.02, 1.90) | 0.05 | 0.04 |
|  |  | BMI | 4.50 (2.60, 6.40) | 0.00 | 0.20 |
|  |  | Sex (Male) | 13.00 (-4.10, 29.00) | 0.14 | 0.02 |
|  |  | Dominant Side (yes) | -1.60 (-18.00, 15.00) | 0.85 | 0.00 |
|  | **Maximum Muscle Force (LSI)** |  |  |  |  |
|  |  | Tendon Retraction | -2.30 (-3.70, -1.00) | 0.00 | 0.12 |
|  |  | Hematoma Size | -3.00 (-4.40, -1.50) | 0.00 | 0.15 |
|  |  | Wood (5) | -7.30 (-19.00, 4.40) | 0.22 | 0.02 |
|  |  | Age | -0.18 (-0.58, 0.22) | 0.37 | 0.01 |
|  |  | BMI | -1.40 (-2.30, -0.59) | 0.00 | 0.11 |
|  |  | Sex (Male) | -5.20 (-12.00, 1.70) | 0.14 | 0.02 |
|  |  | Dominant Side (yes) | 4.60 (-2.10, 11.00) | 0.18 | 0.02 |

**T**able S1. Univariate linear regression of independent pretreatment injury and patient characteristics against dependent Limb Symmetry Indices (LSI) of MRI muscle quality outcome measurements and maximum muscle force. LSI was calculated: value of injured limb/ value of uninjured limb *100. For categorical variables, the category shown in parentheses represents the reference level used in the regression model.

| **Table S2. Model selection with Leaps R package** | | | | | | | | | | | | | |
| --- | --- | --- | --- | --- | --- | --- | --- | --- | --- | --- | --- | --- | --- |
| **Model** | **Age** | **BMI** | **Tendon Retraction** | **Hematoma Size** | **Sex (male)** | | **Dominant injured (yes)** | | **Wood (5)** | | **Adjusted R^2^** | |  |
| **Dependent Variable: Lean Muscle Volume (LSI)** | | | | | | | | | | | | | |
| **6 ( 1 )** | ***** | ***** | ***** | ***** | | ***** | | ***** | |  | | **0.48** | |
| 7 ( 1 ) | * | * | * | * | | * | | * | | * | | 0.47 | |
| 5 ( 1 ) | * | * | * |  | | * | | * | |  | | 0.45 | |
| 6 ( 2 ) | * | * | * |  | | * | | * | | * | | 0.45 | |
| 5 ( 2 ) | * | * | * | * | |  | | * | |  | | 0.45 | |
| 4 ( 1 ) | * | * | * |  | |  | | * | |  | | 0.43 | |
| 4 ( 2 ) | * | * | * | * | |  | |  | |  | | 0.42 | |
| **Dependent Variable: Muscle Fat Fraction (LSI)** | | | | | | | | | | | | | |
| 5 ( 1 ) | * | * | * | * | |  | | * | |  | | 0.48 | |
| 6 ( 1 ) | * | * | * | * | |  | | * | | * | | 0.48 | |
| 4 ( 1 ) | * | * | * | * | |  | |  | |  | | 0.48 | |
| 5 ( 2 ) | * | * | * | * | |  | |  | | * | | 0.48 | |
| **6 ( 2 )** | ***** | ***** | ***** | ***** | | ***** | | ***** | |  | | **0.48** | |
| 7 ( 1 ) | * | * | * | * | | * | | * | | * | | 0.48 | |
| 4 ( 2 ) | * | * | * |  | |  | | * | |  | | 0.47 | |
| **Dependent Variable: Maximum Muscle Force (LSI)** | | | | | | | | | | | | | |
| 5 ( 1 ) | * | * | * | * | |  | | * | |  | | 0.24 | |
| 4 ( 1 ) |  | * | * | * | |  | | * | |  | | 0.24 | |
| **6 ( 1 )** | ***** | ***** | ***** | ***** | | ***** | | ***** | |  | | **0.23** | |
| 5 ( 2 ) |  | * | * | * | | * | | * | |  | | 0.23 | |
| 6 ( 2 ) | * | * | * | * | |  | | * | | * | | 0.23 | |
| 3 ( 1 ) |  | * | * | * | |  | |  | |  | | 0.22 | |
| 7 ( 1 ) | * | * | * | * | | * | | * | | * | | 0.22 | |
| Note: Models are sorted by adjusted R-squared values. | | | | | | | | | | | | | |

**T**able S2. Model Selection Using the Leaps R Package. This table displays the results of a systematic model selection process conducted using the Leaps R package. For each dependent variable, all possible subsets of predictors were evaluated and ranked by their adjusted R-squared values. Candidate models are presented with the predictors included in each model marked by asterisks, and higher adjusted R-squared values indicate greater explanatory power. An nbest value of 2 was used to capture multiple top candidate models, and the model ultimately selected for subsequent analysis is highlighted in bold*.*

| **Table S3. Model Evaluation Metrics** | | | |
| --- | --- | --- | --- |
| **Dependent Variable** | **Adjusted R^2^** | **AIC** | **RMSE** |
| Lean Muscle Volume (LSI) | 0.48 | 701.36 | 13.26 |
| Muscle Fat Fraction (LSI) | 0.48 | 875.55 | 34.28 |
| Maximum Muscle Force (LSI) | 0.23 | 750.12 | 15.10 |
|  | | | |

**T**able S3 summarizes the performance of the final regression models for the three dependent variables. For each model, the adjusted R-squared value indicates the proportion of variance in the outcome explained by the predictors. Lower Akaike Information Criterion (AIC) values signify better model fit, while the Root Mean Square Error (RMSE) reflects the average prediction error.

| **Table S4. Model Diagnostics Tests** | | | | | | |
| --- | --- | --- | --- | --- | --- | --- |
| **Dependent Variable** | **Durbin-Watson** | | **Breusch-Pagan** | | **Shapiro-Wilk** | |
|  | **Statistic** | **P value** | **Statistic** | **P value** | **Statistic** | **P value** |
| Lean Muscle Volume (LSI) | 2.13 | 0.73 | 4.94 | 0.55 | 0.98 | 0.22 |
| Muscle Fat Fraction (LSI) | 2.05 | 0.58 | 11.44 | 0.08 | 0.98 | 0.11 |
| Maximum Muscle Force (LSI) | 1.95 | 0.39 | 5.74 | 0.45 | 0.99 | 0.90 |
| Note: the maximum Variance Inflation Factor (VIF) ranged from 1.01 to 1.37, indicating minimal multicollinearity. | | | | | | |

**T**able S4. This table presents the results of diagnostic tests conducted on the selected regression models for the three outcomes (Lean Muscle Volume, Muscle Fat Fraction, and Maximum Muscle Force). The Durbin-Watson test evaluates autocorrelation in the model residuals. The Breusch-Pagan test examines heteroscedasticity by testing whether the variance of the residuals is constant across observations. The Shapiro-Wilk test assesses the normality of the residuals.

***
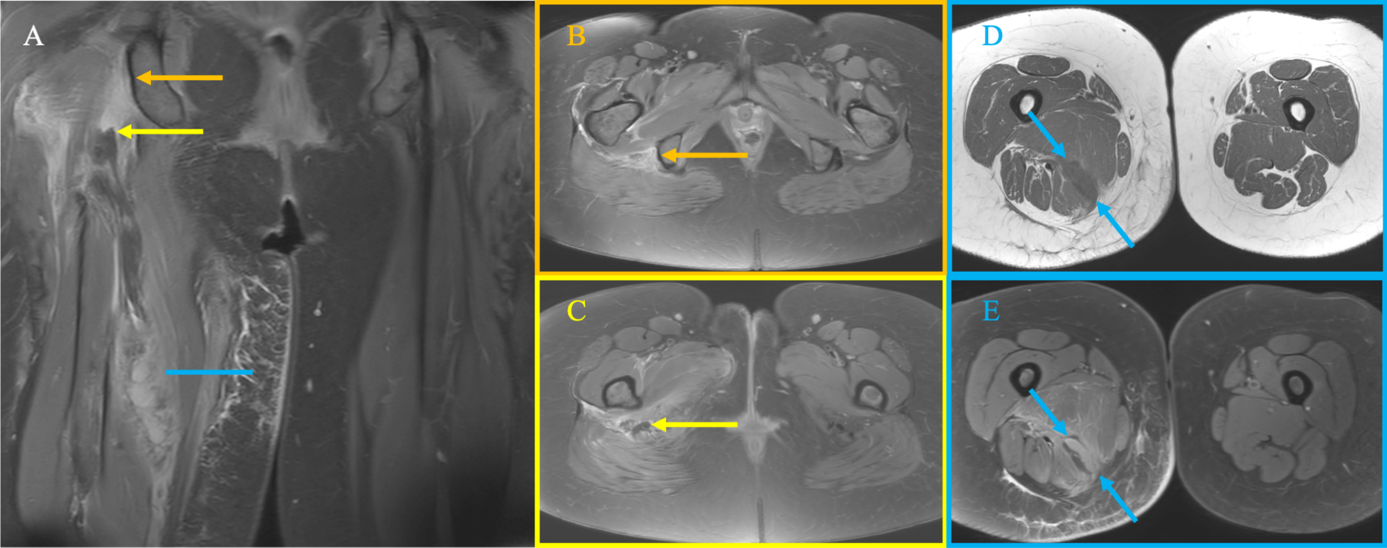
***

**F**igure S1 Coronal PD SPAIR (A), axial PD SPAIR (B, C and E) and axial T1-weighted MR images (D) showing a total avulsion of the right proximal hamstring tendon and a hematoma. Orange arrows pointing at the center of the proximal hamstring complex origin on the upper region of the ischial tuberosity (A, B). Yellow arrows pointing at the most proximal part of the hypointense tendon stump (A, C). Tendon retraction measurement was performed in (A) between the orange and yellow arrowheads. Blue line (A) showing the axial plane (D, E) having the largest hematoma, with blue arrows pointing at the largest hematoma diameter situated between the arrow heads (D, E).

***Supplementary Methods: Dixon MRI Acquisition and Analysis***

All patients were scanned locally at each study site using either 1.5 T or 3 T scanners from General Electric (GE, Milwaukee, USA), Philips (Philips, Best, Netherlands), or Siemens (Siemens Healthineers, Erlangen, Germany). Fat–water–separated images covering the entire lower extremities from the iliac crest to the feet were acquired using a standardized protocol consisting of four overlapping stacks of T1-weighted 2-point 3D Dixon sequences (Lava-Flex [GE], mDixon [Philips], Dixon-VIBE [Siemens]). Each patient was positioned supine, feet first, with surface coils covering the scan area. No cushions or padding were used under the knees or thighs to avoid compression of the musculature of interest. After image acquisition, in-phase (IP), out-of-phase (OP), fat, and water images were uploaded to AMRA Medical (Linköping, Sweden) for analysis.

The standardized scan protocol and a detailed manual were distributed to all imaging sites. After successful protocol installation and remote staff training, each site submitted a certification scan to AMRA for validation of correct protocol implementation and image quality. Upon approval, the site was certified to scan study participants. A central reading system was used, and all images were analyzed using the cloud-based AMRA® Researcher service to ensure comparability across sites.

Detailed descriptions of the complete analysis chain—including scan protocols, image processing, segmentation methods, and quality control measures—have been published previously (1–3). In summary, a proprietary signal calibration converts the images to quantitative fat-concentration maps using subcutaneous fat as an internal reference, making the resulting images comparable across field strengths and Dixon-reconstruction methods (2,4). The calibrated images are then merged into a 3D volume, and the muscles are segmented using an atlas-based segmentation engine. Image quality and automatic segmentations are reviewed by a trained operator and adjusted as needed. After final approval by a second operator, MRI biomarkers are automatically calculated from the segmented regions using the calibrated fat images.

All hamstring muscles (semimembranosus, semitendinosus, and the short and long heads of the biceps femoris) were bilaterally segmented in their entirety.

***APPENDIX REFERENCES***

1. Widholm P, Ahlgren A, Karlsson M, et al. Quantitative muscle analysis in facioscapulohumeral muscular dystrophy using whole‐body fat‐referenced MRI: Protocol development, multicenter feasibility, and repeatability. Muscle Nerve 2022;66(2):183–92.

2. Borga M, Ahlgren A, Romu T, Widholm P, Leinhard OD, West J. Reproducibility and repeatability of MRI‐based body composition analysis. Magn Reson Med 2020;84(6):3146–56.

3. Karlsson A, Rosander J, Romu T, et al. Automatic and quantitative assessment of regional muscle volume by multi‐atlas segmentation using whole‐body water–fat MRI. J Magn Reson Imaging 2015;41(6):1558–69.

4. Karlsson A, Peolsson A, Romu T, et al. The effect on precision and T1 bias comparing two flip angles when estimating muscle fat infiltration using fat‐referenced chemical shift‐encoded imaging. NMR Biomed 2021;34(11):e4581.
